# Medical Jurisprudence

**Published:** 1829

**Authors:** 


Art. 6.—Medical Jurisprudence.—*Report of a trial for Murder, in which the culprit was defended on the ground of his labouring under Mania a potu, or Delirium from Intemperance.* By the Editor.

James Birdsell, of the village of Harrison, in the western part of this county, on the 3d inst. was arraigned before the Supreme Court of Ohio, for homocide. Present on the bench, Judges Pease and Sherman. In the indictment it was laid, that the defendant, on Thursday evening, 5th of March, 1829, murdered his wife, by cutting through her neck from side to side, with a narrow axe, at a single blow, which severed the spinal column, and caused instant death. The proof of the fact was perfect and uncontroverted. The defence set up was *Mania a potu.* Having recorded the principal facts, and also been furnished with the notes of the prosecutor and one of the advocates, I propose to lay before the reader such portions of them, as have a relation to the subject of *Medical Jurisprudence.*

The defendant was to appearance about fifty years of age, and had been married nineteen or twenty years to a second wife, by whom he had several children, one of whom was a witness in the case. It appeared from the testimony, that for several years he had been subject to occasional fits of intoxication, which in the latter part of the time had been followed by *Mania a potu*, which generally lasted for several days, and went off spontaneously. In these paroxysms he had the physical and moral symptoms which usually characterize that malady. The former were, great tremors of the hands, a pale face, red eyes, and sometimes a copious perspiration even when exposed half naked to a cold atmosphere. The moral phenomena were, disordered perceptions of sight and hearing, so that he often insisted, that he saw himself surrounded by snakes and other reptiles, or by armed men who sought to kill him; or supposed he heard strange sounds of trumpets, or vocal music, or conversation of which he was the subject, and the object of which was mischief to himself. He was thus filled with apprehension for his safety, and sometimes ran about the village at night, as if attempting to escape from bad persons who were pursuing him. On a certain night, he made so much clamour as to excite the idea of several men engaged in a riot. At another time, in his own house, he concealed himself between the feather and the straw bed, where he was almost suffocated. On another occasion, he was found, after dark, standing in the street without shoes or hat, and had described around him a circle in the dust, and declared, that if any one entered it, that person would kill him. At other times he would peep from his window, and point his gun, as for defence, against imaginary persons, who were approaching to seize him. Again, he would fancy that two armies were engaged in battle, and that he must join one of them. In all his paroxysms he had so great a degree of watchfulness, as to sleep little or none for several nights in succession. But his prevailing maniacal conception was, that his wife was in a combination with three of his neighbours, one of whom was his son by a former wife, and that they had conspired against his life. Of these men, when they were not in his presence, he was afraid. In the paroxysms he was accustomed to charge his wife (unfoundedly in the opinion of witnesses) with a criminal intimacy with these persons. He even threatened to kill her if she did not desist, and had been heard to utter this threat, when he was thought by one of the witnesses to be rational.

On the Sunday before the murder, he drank freely, and was intoxicated, in which condition, as usual, he was quiet, dull, and disposed to lie in bed. Monday, Tuesday, and Wednesday presented nothing special. On Wednesday evening he complained to a neighbour of feeling unwell, and asked his son's assistance in the performance of some necessary manual labour for his family. He seemed to the witness to be rational. During the night he slept none, and complained of cramp in his stomach. The next morning his family thought him crazy, but were not alarmed, as they were accustomed to such attacks. In the course of the day, he took an axe on the shoulder, and walked rapidly to the house of a neighbour, whom he desired to go home with him, saying they wanted to kill him; and about the same time he told another of the supposed conspirators, that he had overheard his wife and him that morning whispering about taking his (the witness') life.

He spent the day at home in the midst of his family, apparently in agitation and terror, but said he would not hurt any one, and did not wish to be hurt. In addition to the axe, which he placed under the bed, where it was often kept, he provided a scythe, which he brought into the house. He manifested jealousy of his wife, and told her to act better, for she had already caused the death of 30,000 men. He fancied that the persons of whom he was jealous, were in the loft manufacturing ropes to hang him, and going up, returned and said he had cut the ropes to pieces, and brought down the fragments with him, though he had nothing in his hands. In the course of the afternoon, he fastened both the doors of his house. At the usual time his wife went out to milk, and he barred the door after her. On her return he fastened it again. She was seated near the fire, and he was walking the room. At length he took the axe from under the bed, and suddenly gave the fatal blow, following it up with two others on the face. His oldest daughter caught the instrument, which he yielded up, and then seized the scythe, with which he attempted to strike her. She defended herself with a chair, till the smaller children having opened the door, she made her escape. He took his youngest child in his arms, and sat down by the window. The child exclaimed, 'mamma bleeds!' which he said made him feel bad. When his neighbours arrived immediately afterwards, he gave himself up, acknowledged what he had done, said he knew he would be hung for it, but that he ought to have done it nine months sooner; and that if he had it to do again, he would strike two blows where he only struck one. Talked so rationally, that many of the witnesses could not believe him deranged. Evinced no dread of punishment for his crime, but was still in great apprehension from the persons who, he had believed, intended to kill him. Was glad that he had defeated their calculations. On his way to the city to be committed to jail, talked rationally and composedly about his affairs, and on various subjects; but frequently asked the guard if they did not hear sweet sounds of different kinds, and on being answered in the negative, insisted that he could not be mistaken. After being committed he became regular, and expressed his regret at what he had done.

Three medical gentlemen were required to deliver professional opinions on the testimony. They came to the same conclusion, which was, that the defendant was subject to *Mania a potu*, and that he laboured under that malady when he perpetrated the homicide.

On this opinion, and the facts which support it, the counsel for the prisoner rested his defence. It was alledged, that *Mania a potu* should not be confounded with the delirium of a fit of intoxication; but associated with insanity, and, like it, might be offered as a legal excuse for the perpetration of what, in its nature, is felonious. They admitted, that drunkenness cannot be plead as an apology for crimes, but argued, that when it gives birth to insanity, the maniac should experience all the immunities of a *non compos* from any other cause. They averred, that if a man, in the commission of a felonious deed, should receive a blow on his head, which was followed by mental alienation, his acts, during that state, would not be regarded as criminal; and that the subject of *Mania a potu* is in that condition; lastly, that the habitual drunkard is urged on by a physical necessity which he cannot resist; and should not, therefore, be held accountable for his conduct when labouring under the derangement, which follows his inebriation.

To which the prosecutor replied, that *Mania a potu* should be referred to drunkenness rather than insanity; that it follows as the necessary consequence of the former, and soon abates; that the defendant, Birdsell, was aware that his intoxication was productive of delirium, and, hence, that he *voluntarily* did what he knew would derange his intellect; that *Mania a potu* being a natural effect of intemperance, cannot be offered as an excuse, like the insanity which might follow a blow on the head, received during the perpetration of a felony; which blow and insanity should be regarded as mere contingent or accidental events; finally, that the law does not recognise, in the drunkard, a *physical necessity* to drink; but holds him accountable for his actions when intoxicated; and, of course, in the delirium which follows that intoxication.

The opinion of the court was looked for with anxiety; but in their charge, they waived a discussion of the point in controversy, and submitted it, with the facts, to the jury, who returned a verdict of murder in the first degree, as laid in the indictment.

---

It is certainly a matter for regret, that the highest tribunal of the state, should not have delivered an opinion on the law in this case. The question was presented to them detached and unincumbered. A more favourable opportunity cannot again occur. From the prevalence of *Mania a potu*, it is most desirable, that its character in relation to our criminal code, should be ascertained. If the Court could not say what the law is, they might have said what it ought to be. One step, on ground which must sooner or later be travelled over, would thus have been taken, and that, too, by jurists of acknowledged ability.

Till within the last twenty years, *Mania a potu* had received but little attention as a subject of practical medicine; and, in reference to medical Jurisprudence, it had attracted still less. In his able treatise on insanity according to the laws of

England, Dr. Haslam evinces, that even he has ideas on this subject not altogether correct. In a note he uses the following language:—

A broad distinction should be made between the immediate and remote effects of intoxication. A man is not held guiltless who perpetrates a crime during the state of intoxication. He *voluntarily* introduces into his system a stimulus which augments his ferocity, diminishes his moral affections, and overshadows his reason. But the usual effects of this stimulus is temporary, he awakes from his debauch, rational, and commonly drags after him the heavy chain of reflection. It is however equally true that this single excess may be continued into permanent insanity: he may remain for many months in a state of mental derangement, and during the prevalence of his disorder may be compelled to forego all intoxicating beverage.—If such person after the lapse of several weeks from the commencement of his disorder, should, under its influence, commit a fatal outrage, no system of jurisprudence would connect the violence with the cause which originally produced the disease.

*Cooper's Tracts on Med. Jurispr. p.* 317.

Now I am inclined to think, that *Mania a potu* is never *continued* into permanent insanity, except the individual be *predisposed* to that malady, when it may be excited by various causes, both moral and physical, and among others by intemperance; but this is not *Mania a potu.* That malady is always transient—often continuing for two or three days, only, and seldom extending beyond a fortnight. Like mania, and melancholy or monomania, it exhibits, in different cases, every degree of hallucination, from the perception of a single imaginary object, up to throngs so complicated and fantastical, as to absorb the whole attention and controul all the actions of the individual. Some patients are quite conscious that the whole is unreal; but in others the belief in the reality of their disordered conceptions is of the firmest kind. The power of combining these conceptions, is sometimes wanting: at other times they are connected like the ideas of the monomaniac, and the individual exhibits a fixed purpose with associated ways and means for its accomplishment. Now the question is, whether during a paroxysm of this kind, the individual, in proportion to the degree of his alienation, should be held exempt from the penalties of the criminal law?

On this question jurists, physicians and moralists are divided. They all agree, however, that if *Mania a potu* should continue for months, or even weeks, the individual would be irresponsible. But it might be asked why more irresponsible at the end of many weeks, than at the beginning of the first? If two drunkards should be seized with *Mania a potu*, at the same time, and on the first day, one of them should perpetrate a felony, and the other do the same at the end of thirty or sixty days, on what principle of justice, should the former be punished and the latter pardoned? His more protracted delirium might even be regarded, as *prima facie* evidence of greater intemperance, and consequently of greater depravity; for the criminality of the acts of a person affected with *Mania a potu*, can only be deduced from the criminality of its remote cause—drunkenness. Tested by a reference to the origin, nature and consequences of their delusion, the two individuals present an exact identity. They differ only as to the stage of the delirium in which they commit their respective felonies. But a difference in *time*, like a difference in *degree*, does not constitute a difference in *kind;* and what is criminal in one, cannot be innocent in the other. No jurist or legislator could venture to assert, that because, in one case, the crime was committed sooner after the intoxication than in the other, it was, *therefore*, more felonious. In both cases, the intoxication has ceased. A new and distinct delirium has arisen; which affecting both individuals alike, renders them alike irresponsible to the law, or leaves them equally subject to its penalties. It is to be regretted that so many persons confound drunkenness with its consequence, *Mania a potu.* All who do this, are liable to fall into the absurdity, of holding the maniac more accountable on the first, than the last days of his insanity; when, throughout the whole period, his actions should be brought to the same standard, unless it can be shown by testimony, that his delusion has increased or diminished.

But let us recur to the question, whether *Mania a potu*, either temporary or protracted, should destroy accountability to the criminal law? If the subject of this malady be held responsible, it must be either, because his delusion, originating remotely in a viscious habit, partakes of that character; or, because it does not destroy the perception of right and wrong.

First. Those who believe that *protracted Mania a potu* should render the individual irresponsible to the law, must as I have shown, regard him as exempt from its penalties from the beginning; and as a great majority, in society, are probably of that belief, we might presume that the question is already decided in favor of the maniac from drunkenness. There are not a few, however, who, on being presented with the alternative of punishing or excusing, *all* his maniacal outrages, consider it just and necessary to adopt the former.—Regarding intemperance as voluntary and criminal, and considering *Mania a potu* as its legitimate effect, they would apply the same law equally to every part of the drunkard's conduct, from the commencement of his fit of intoxication, down to the utter overthrow of social feeling, moral principle, and sound intellect, which characterize his mental alienation. The cause of temperance and the safety of society, say they, alike demand this severity. It will deter others from drunkenness, and of course protect society, by anticipation, from the enormities which follow in the train of that vice. If it would, indeed, work out this salutary effect, we might be justifiable in punishing *non compos*, for the crimes which he unconsciously commits. But I cannot bring myself to believe, that this desirable end can be thus attained.—Few men, perhaps none, when presented with the prevailing temptations to drunkenness, are likely to anticipate consequences so remote, or contemplating, will be deterred by them. I even doubt, whether the known responsibility of the drunkard to the criminal and civil law, for all his conduct during a fit of intoxication, contributes in any great degree, to repress intemperance. Its chief influence, seems to me to consist in preventing bad men from real or simulated intoxication, as an excuse for the crimes which they desire to perpetrate. In this lies the wisdom of the law, and the necessity for its existence.

Drunkenness in all its effects—present and prospective—is a terrific moral evil, which every man should labor to suppress. But we must work with other instruments than the halter and the gallows. No criminal code ever succeeded by its cruelty. To cut off dangerous men from society is comparatively easy: to prevent their reproduction is the task. A bad government limits itself to the former, but a good one seeks to accomplish the latter. Its great object is reform, which it effects by education—by removing temptation—by restraints and disabilities—by certain but mild penalties. Its eye is sleepless, but never ferocious. It feels no malice, indulges in no revenge, and delights not in the sufferings which it inflicts. Its chastisements are tempered with mercy, and its decrees of death not written in blood but the milk of human kindness.

It is notorious that many good men fall into habits of intemperance. The insiduous influence of example, inordinate appetites, bad health, disasters in business, domestic misfortunes, or private griefs, decoy or jostle them from the long trodden paths of sobriety, to which many of them never return. Suppose a man of this class—one for example who had experienced some great misfortune in trade—to become at length the subject of *Mania a potu*, and murder his wife—the woman whose participation in his misfortunes had armed them with the stings of scorpions, and led him to seek relief in the use of ardent spirits;—should *such* a man be ranked with criminals and condemned to death?—inflicting on his innocent orphans a new and more terrible anguish—on their very name a disgrace which all their tears could not wash away? This may be called a strong case, but it tests the principle. If one man is punishable for his actions when laboring under *Mania a potu*, all others are equally obnoxious, as the law should not go back to the causes of intemperance, and measure out its punishments according to their relative crimininality.

We come, in the second place, to inquire whether the delirium in *Mania a potu*, is of that *kind* which should secure the individual from the penalties of the law.

The recorded cases of this disease, together with those which I have seen, leave little doubt in my mind, that this question ought to be answered in the affirmative. Like the other forms of mental alienation it occurs with some diversity in its symptoms, and with much variety in its degrees. Now, there are two principles of judicial restriction, which are applicable to all kinds of insanity. First. They entitle *non compos* to immunity from punishment but in proportion to their degree. Therefore when his morbid conceptions are not very vivid, and his power of judging is but little impaired, his claims on the clemency of the law are weaker, than when his malady is more intense. Secondly. When he is deranged on one subject only, his insanity cannot be given in excuse for acts which are foreign to that subject.

Hence it does not follow as a matter of course that mental alienation will justify every felony. It must be shown, either that the culprit is insane on all subjects, to such a degree as to pervert his affections and destroy his estimate of right and wrong; or that his crime had some natural connexion with the subject of his hallucination. *Submitting to these requirements*, the subjects of *Mania a potu* seem fairly entitled to plead their alienation, when arraigned for crimes. As I have already said, they have disordered conceptions, of intense and bewildering vividness, which, it is true, they sometimes regard as delusions, but oftener mistake for realities, and act accordingly. Their passions, particularly those of fear, jealousy and anger, have an uncontrollable mobility; their desires and aversions are equally morbid, and their will displays a wild and sleepless energy of action. In this condition, they may still distinguish between right and wrong, on many subjects, provided their attention can be fixed, and the subjects have no connexion with the trains of thought with which they are incessantly occupied; but in reference to these, they judge and act on the conviction of their reality; that is, with, a firm belief, and should not, therefore, be held accountable. Thus in the case of Birdsell, the predominant opinion, in all his paroxysms, was, that two of his neighbours, and his own son, had conspired against his life: and would, after they had killed him, unite themselves with his wife, whom he also considered in the conspiracy. Under this delusion he killed her, determined, as he said, 'to defeat their calculations.'

Notwithstanding this dreadful catastrophe, it cannot be said on the whole, that *Mania a potu* is one of the most dangerous forms of mental alienation, especially in old inebriates, whose constitutions are broken down. Most of them are cowards, and far more disposed to flee from danger, either real or imaginary, than to face it. Indeed, terror is, in some degree, the characteristic of this form of insanity, and has shown itself in many striking and ridiculous modes of action; but fear is not incompatible with the perpetration of outrages, when *non compos* thinks he can strike a blow, which may lessen the danger that he believes to surround him.

But should society interfere with the subjects of *Mania a potu?* when that malady in general lasts but a few days, when in many cases the patient is ill, and in others the delirium would pass off before any complicated system of measures could be executed? I answer yes; for in that brief period, acts the most horrible may, possibly, be perpetrated; and on this possibility, rests the duty of our interposition.

Ought writs of *lunatico inquirendo*, under the existing statute, be sued out against persons in this condition? This would certainly be preferable to suffering them to go at large; but the regulations for protracted or permanent insanity, do not seem to be well adapted to a temporary delirium, and are not likely to be enforced.

I am of opinion, that the case calls for special legislation; but shall not presume to insist on the mode, in which the power of the state should be exerted.

In regard to drunkenness itself, our laws require to be revised and extended. If the drunkard be allowed his personal liberty, he ought to be subjected to several restrictions and disabilities, such as excluding him from juries, and from all civil offices; diminishing his credibility as a witness; depriving him of the elective franchise; and consigning his property to trustees. By these measures, not at all inconsistent with the genius of a mild and beneficent system of jurisprudence, much might be accomplished in the prevention of drunkenness, and, consequently, of *Mania a potu.* Whenever that complaint might occur, however, the individual should be apprehended and confined, not at the discretion of friends, but in obedience to a law that should admit of no exceptions; and at the same time (if his property had not been previously subjected to trustees) that step should be taken. The disgrace and mortification attendant on such a proceeding, would do more to deter men from intemperance, than a multitude of public executions; while it would protect families and the community from the outrages which it is the object of the present system to punish, rather than prevent.

Since the above was sent to the press, a motion for a new trial has been argued. Harrison and Drake for the prisoner—Wade for the commonwealth. The Court, having deliberated on the application, refused to interfere with the verdict of the jury. I had not the advantage of being present when their opinion was delivered, but understand that they said, that the question whether Birdsell, at the time when he murdered his wife, had the power of discriminating between right and wrong, was one of fact, to be made out by evidence, and properly within the province of the jury. The decision having been made by that tribunal, they felt no disposition, nor did they see any good reason, for disturbing the verdict.

Upon the question, whether *Mania a potu* could, under any circumstances, be given as an excuse for the commission of a crime, they were not called upon, in the present case, to decide. They, however, felt no unwillingness to express their opinion, that if the insanity was the offspring of intemperance, and the prisoner *knew* that intoxication would produce it, he could not plead it as an apology. I have endeavoured, unsuccessfully, to learn, whether the court turned the responsibility of the defendant, upon the fact of his *knowing* that derangement would follow his drunkenness; or upon the moral turpitude of that vice, by which it imparts to all its consequences a character of criminality. I am likewise unable to ascertain, whether the jury convicted the prisoner, because, in their opinions, his insanity was not made out; because the act which he perpetrated was not connected with his disease; or because they would not allow insanity, from drink, to constitute an excuse. The general impression is, that both the court and jury held, that Birdsell was not deranged in the degree that destroyed his perception of right and wrong, in reference to the murder; and that, if he had been, still he could not have been acquitted, because his alienation originated in intemperance.

It is not without hesitation, and a sentiment of déference, that I venture to dissent from both opinions. It seems to me, that the insanity of the culprit *was* sufficiently established; and that he was not capable of distinguishing between right and wrong, or at least of controlling his actions, on the subject of his hallucination. That subject was, the supposed infidelity of his wife, and her collusion with his *son*, and two of his neighbors, who conspired against his own life. In all his maniacal paroxysms, these ideas occupied his attention, and were conspicuously exhibited in the last attack. On Sunday he was intoxicated, but afterwards drank nothing. On Monday, Tuesday, and Wednesday, he was sober, and seems to have been regular. On Wednesday night he became sleepless and unwell; on Thursday morning he took his axe, and went off rapidly to one of his neighbours, requesting assistance, for they were going to kill him; he returned and spent the day in upbraiding his wife, and declaring that her supposed confederates were concealed about the house, making ropes to hang him; he barred the doors; when she went out in the evening he barred them after her; on her return, his conviction still continuing, he seized one of the instruments of defence which he had provided, and as she was sitting, unalarmed, by the fire, did that which was perfectly consistent with his false impressions,—put an end to her existence, as a punishment for her supposed criminal attachment to others, and to dissolve their conspiracy against his life. His daughter attempted to arrest his arm, which, in his estimation, brought *her* into the combination, and he aimed a blow at her. The murder being perpetrated, he made no attempt to escape; avowed and justified the act, but said he expected to be hung for it; came willingly with the officer to the city; at times heard sweet sounds of music on the way, but at others talked of the canal which they travelled along, and of other common matters, with great composure, and perfect rationality. Deposited in jail, he soon manifested no further symptoms of mental alienation.

Now, I appeal to the enlightened members of the profession every where, whether here are not all requisites of a case of monomania:—Assumed and unreal premises,—deductions true to the principles of logic, but false in point of fact,—lastly, acts consistent with his conclusions. On other subjects he might have been regular enough, but on this he had gone estray; on others, he could judge between right and wrong; on this, his estimate of right and wrong took its character from his delusion. Had he killed, in a real dispute, some other person, not of the conspiracy, the act would have been foreign from his hallucination, and should not have been excused; but when he murdered one of the conspirators, he kept himself within the pale of that hallucination, and acted in accordance with his erroneous conceptions. Both the Court and prosecutor admitted, that he laboured under derangement; but because he manifested a perception of right and wrong on certain subjects, seemed disposed to argue, that he must have had a perception of right and wrong in reference to the subject of his insanity! I would ask, whether if he had been equally insane on all other topics, they would have looked for manifestations of a correct estimate of right and wrong? I think they would not, and still, to be consistent, they should.

It must not be forgotten, that it was admitted by all, that he was deranged. The difference of opinion related to its degree,—the prosecutor and others, regarding it as insufficient to justify him, others as sufficient. But he did an act, felonious in its nature, directly connected with his erroneous opinions; and I would put to them the question, whether, if he performed a sane action, and for such only could he be held accountable, it would be one connected with his insanity? I think not. *Non compos* cannot be of sound mind on the subject of his derangement, though he may be on every other.

I will here take occasion to say, that of the various forms of mental alienation, monomania, or partial insanity,* is not only the most dangerous to the peace of society, but by far most difficult to establish to the satisfaction of an ordinary jury. The broad and hideous features of *general* madness, or mania proper, cannot be mistaken by the most ignorant; but such madmen are generally harmless. They may give an unlucky blow, but they have no design, no plan, no perseverance, no concealment. Not so with the monomaniac. On some subject, or group of subjects, his conceptions are wrong, and his assumptions false. The error lies in his premises, more than his conclusions; you may reason him into a modification of the latter, by correcting his logic: but no argument, persuasion, or authority, can drive him from the former. The spirit of his delusion cannot be exorcised. It governs him with despotic power, but its jurisdiction is limited. He has, as it were, two cotemporary states of intellectual being, with alternate manifestations; one in common with those around him; the other peculiar to himself. In the former, he feels, and thinks, and acts, in consonance and sympathy with his race; in the latter, he contemplates nothing but the objects embraced in his delusion; and regardless of consequences, looks only to their accomplishment. In his morbid state, we may often see much of his original character. If mild, timid, and benevolent, his insanity is less to be dreaded—if suspicious, he becomes jealous—if proud, haughty and impetuous—if ill natured, ferocious and revengeful. Hence, men of bad passions are most likely, when insane, to commit outrages, and are least likely to receive the commiseration of society, or the courts of justice, which seems to have been the character and fate of the convict Birdsell. But whatever may have been the natural disposition of the monomaniac, or the nature of his delusion, it can never be pronounced absolutely safe, to allow him unrestrained liberty. His hallucination may con-

*It may not be amiss for us to recollect, that a *partial* insanity is not an alienation slight in degree, but limited in the extent of its objects. Under this view a derangement may be on a single point only, and still have a character of most terrific energy.

tinue the same, but the actions which it originates, may vary from day to day. He may alter his plan of operations, while his objects remain unchanged. Passions the most opposite may possess him in successive hours. The flame which lights them up is not from without, but lies smouldering in the recesses of his own bosom. Hence his conduct cannot be foretold. He sets prediction at defiance, and at the moment when the watchmen around him cry 'all's well,' he may be meditating outrages of the bloodiest die.

Since I began this paper, I have more than once laid down my pen, to listen to the story of a monomaniac. Five or six days ago a stranger applied to me for professional advice, and recounted so many bad symptoms, as at first to astonish me. At length I asked him the cause of his indisposition, and he told me with a low and earnest voice, that he had been poisoned by a woman in a distant state. Suspecting insanity, I advised him to take a warm bath, to promote the expulsion of the poison through the skin, and call on me again. Three days afterwards he joined me in the street, and informed me, with great concern, that the woman who had poisoned him, was now on her way up the Ohio river, and would certainly take his life. I said, I hoped not. But, said he, how can it be prevented? Let me know, I replied, when she arrives, and I will protect you. But, how *can* you, he asked, she works secretly? I must not tell you, said I, or it will take away my power. This seemed to increase his confidence, and with many expressions of gratitude, he left me to watch the arrival of the steam-boat. The next day he came to my office, in a state of increased alarm, to inform me, again, that she was hourly expected; that her husband was with her, and that lodgings had been provided for them in a certain part of the city, which he described; that she was considered a respectable woman, in society, but had killed several men; that he had loved and addressed her, but was superseded by the richer man, to whom she is now married; that she first gave him arsenic in an apple, and afterwards poison on a rose, which had penetrated all parts of his body, except his heart, which was only protected and his life saved, by wearing over it a charm cut out of paper; that when she arrived, she would certainly kill him, and afterwards *must* kill her husband, because of their having addressed her as rivals. At length he declared, that if he should see her in the street, he would take her life. But, said I, that would be wicked; to which he replied, should I not do it to save my own life? That is true, I remarked, but you would be taken up and hung, and you had better die by her hand than that of the hang-man. This seemed to confound him, but not to quiet his alarm. I succeeded, however, in composing him, by renewed assurances of my power to countervail her designs, even without seeing her, provided I could know when she arrived; and he left me for the night, to watch her coming. The next morning he returned to tell me in a whispering and agitated voice, that she would be at the quay in half an hour, accompanied by two negroes as accomplices, and begged me to be prepared; I renewed my assurances, and advised him to be quiet and keep out of her reach. At noon, he came to me in the street, to say, that he had watched throughout the morning, but she had not yet arrived;—since which, one night has elapsed, and I have not seen him. This unfortunate man has an agreeable but anxious countenance, dresses decently, possesses some intelligence, and converses well when his attention can be diverted from the narrative which I have given. I have no doubt that he is deranged. The story of the expected arrival of a woman may be an illusion; but suppose it real, and that he believes her in quest of him to take his life, after having twice attempted it already, would it be either remarkable or criminal, if he should kill her? It certainly would not; and yet if he were to do so, I apprehend that according to the proofs of madness required in the case of Birdsell, he would be punished.

Should I even sue out a writ of lunacy against him, it is not probable that the court would sustain it, seeing that the proofs of his madness are not so strong as those adduced on the trial of Birdsell,—which were held to be insufficient. And here I will take occasion to remark, that far less evidence of insanity might justify a court in granting a new trial, than would warrant a jury in finally acquitting the prisoner, on the ground of his being *non compos mentis.* A new trial to Birdsell could not have prejudiced society. The fact of the murder could always have been established. If testimony should have been lost, it would have been that which went to prove his madness, and such a loss would have injured him, not the commonwealth.

I am aware that *Mania a potu* often presents only the phenomena of false and disassociated perceptions, like febrile delirium; but this is not always the case. The histories of it, which have been published, have been generally drawn up with a reference to the practice of physic, rather than of jurisprudence; and, therefore, much has no doubt been omitted, that would go to show ,in different cases, some kind of specific delusion in the midst of the general desolation.—Whether it partake most, however, of delirium, of mania, or of monomania, the person affected cannot be regarded as of sound mind.

But dismissing this subject let us inquire into the validity of the opinion that *Mania a potu* cannot be plead as an excuse for actions criminal *per se.* I have already discussed this subject at some length, but shall venture to resume it. If this variety of mental alienation cannot constitute a valid defence, it can only be from the character of its remote cause. But I would ask, whether the court and jury have a right to travel behind the testimony which establishes the insanity, to inquire into its causes, and estimate the culpability of *non compos,* not by the degree of his alienation, but the criminality of those causes? I think they have no such right. But if it is correct for them to do it in one case, it is equally so in all others; and whenever insanity is offered in defence, its causes should be ascertained and made to determine the guilt of the accused. This I apprehend, would be a new principle in jurisprudence. Let us look at the practice to which it would lead. *Mania a potu* is, sometimes, the consequence of the use of opium; and frequently results from daily stimulation with ardent spirits, without their being ever taken to the extent of intoxication.

Now all the acts of a *non compos* from either of these causes, must be pardoned, because there is nothing criminal in such use of stimulants. Moreover, drunkenness itself is not unlawful, and cannot, therefore, impart a character of criminality to the actions of him in whom it may excite insanity. There are, however, many other causes of this malady which *are* criminal, such as gambling, duelling and prostitution, all of which should be inquired into, and when found real, must, if the principle is adhered to, be made to impart criminality to the actions of *non compos*; while he would be exempt from punishment, if his derangement arose from causes not immoral. But this, I venture to assert, was never done in any country. The truth is, that the immunity from punishment, results from the insanity itself, and not from the nature of the causes which produced it. Further, although *Mania a potu* is essentially a temporary disease, it often happens, where there is a predisposition, that insanity is permanently established by intemperance. Now, in such a case, referring to its exciting cause, the individual should be held responsible, throughout the whole of his after life; but the law expressly exempts him from all punishment, save the confinement which his own personal safety, not less than that of society, imperiously requires.

But what shall be said of the doctrine, that the criminality of a *non compos* from drink, lies in his *knowing* that his intoxication will be followed by insanity? The proposition at first view seems plausible, but some difficulties, both in theory and practice, hang about it.

The bad acts of the first paroxysm would thus be separated from those of all the subsequent paroxysms. *Legally* they would be innocent, while the same conduct, in future attacks, would be criminal. Again, all maniacs are not conscious of their insanity after it has passed off, and although they might be told that they had been insane, it by no means follows that they could believe it. Further, although the drunkard who has never had *Mania a potu*, cannot forsee, that it will come upon him, and must, therefore, under this doctrine, be held excusable for felonies, yet it is undeniable, that he knows the general fact, that intemperance is apt to occasion madness; and how slight is the shade of difference between that knowledge, and the knowledge of him, who is subject to *Mania a potu!* They pursue the *same* practices, one with the information that he may *possibly* be made insane, the other with the conviction that he will *probably* fall into that state. I cannot perceive in the two cases a metaphysical or moral difference, sufficient to justify a legal distinction.

A great variety of causes are *known*, among other sinister effects, to produce insanity. Suppose an individual do not avoid these causes, is he *therefore* to be held accountable when he becomes insane? This will scarcely be contended for, and yet it might be found difficult for the ablest judge to chalk out a line of demarcation. Hence I am disposed to doubt whether the doctrine ought not to be abandoned. I perceive no moral or legal necessity for its retention. There is no previous malice towards those who may happen to suffer from the acts of a *non compos;* he does not subject himself to the causes of insanity, for the *purpose* of inducing the malady, that he may under its protection commit a felony; and after his insanity has arisen, he is incapable, in a legal or moral view, of acting with malice. I am, therefore, led to question the soundness of the Massachusetts decision, which condemned M'Donough for the murder of his wife; notwithstanding it has received the approbation of such an eminent medico-jurist as Dr. Beck.* It appears that the wound on M'Donough's head, was of itself sufficient to bring on paroxysms of insanity, but that when they were pending, intoxication instantly excited them. In one of these fits he murdered his wife. He could not have foreseen this, or any other criminal action; he did not, therefore, drink with malice prepense; and his crime seems to have been, merely that he did not refrain from that which promoted the recurrence of the paroxysms, but involved no malice, and is not prohibited by our criminal law. Suppose he had killed her in one of the paroxysms which came on without previous intoxication? The

* Medical Jurisprudence, Vol. 2, p. 375.

court would not, then, have condemned him; but the event might as well have happened in such a paroxysm as any other. When the law makes malice aforethought, indispensable to the crime of murder, a homicide committed by *non compos without* such malice, cannot constitute that offence.

The law however, does not excuse him, who unintentionally commits a criminal act, when attempting to perpetrate a felony of a different kind. The evil design of the first act, imparts criminality to the second, although it may be a mere casualty. But where is the malice or the evil intention of the drunkard? When M'Donough and Birdsell drank to excess, was it with any felonious intention, or did they violate the law of the land? Moreover, the mental alienation which is called *Mania a potu*, comes on after the individual has ceased to drink; and, therefore, if drinking were felonious, the acts of the maniac could scarcely be said to derive criminality from it. If a robber by *accident* kills the person whom he robs, or any other, while in the perpetration of the deed, he is guilty of murder, but if he return home with his booty, and the owner, instead of resorting to the law, should subsequently invade the dwelling of the thief, to recover his property, and should be killed, it would not be murder.

It has been said, that if the drunkard were not held responsible for his doings when affected with *Mania a potu*, he would only have to feign that malady, to escape punishment for all the crimes he might desire to commit. But drunkards are far from being well qualified to simulate madness. Their feelings and judgment are in general too much impaired, to admit of their executing such a delicate and difficult task. Moreover, the strongly marked constitutional or bodily disorders, which attend on *Mania a potu*, could not be produced at the will of the individual, nor by any means which he might employ for that purpose. But why provide so carefully against feigned insanity in drunkards (who are generally incapable of such an act) and leave all the bad men of society at liberty to feign madness, as a cover for their crimes? To be consistent, the law should declare, that real insanity shall no longer be plead in excuse, because pretended insanity has sometimes led to an acquittal of the guilty.

I shall conclude, by expressing the hope, that the case of Birdsell may incite our physicians and lawyers, to a closer study of mental alienation, particularly of *Mania a potu*, in reference to the laws of the different states. It is undeniable that the best of us have many incorrect opinions on this difficult subject: opinions which we owe it to the learned professions of which we are members, and to society at large, to correct as soon as may be convenient. In the observations which I have hastily thrown together, there are no doubt many inaccuracies both of thought and expression, but I hope they will promote inquiry, and having that effect, they cannot fail to do some good.

P. S. Desirous of collecting and publishing authentic cases of *Mania a potu*, drawn up with a reference to Medical Jurisprudence, and consequently containing an accurate account of the mental phenomena, the Editor begs leave respectfully to solicit histories of that kind.

*Erratum.* There is a mistake in the baptismal name of Birdsell, the subject of the foregoing paper; it should be John instead of James.

